# Simulation and Experimental Investigation on Additive Manufacturing of Highly Dense Pure Tungsten by Laser Powder Bed Fusion

**DOI:** 10.3390/ma17163966

**Published:** 2024-08-09

**Authors:** Enwei Qin, Wenli Li, Hongzhi Zhou, Chengwei Liu, Shuhui Wu, Gaolian Shi

**Affiliations:** 1 College of Intelligent Equipment, Suzhou Institute of Industrial Technology, Suzhou 215104, China; qinew@siit.edu.cn (E.Q.); shigl@siit.edu.cn (G.S.); 2 Suzhou Nuclear Power Research Institute, Suzhou 215004, China; liuchengdwei@cgnpc.com.cn (C.L.); wushuhui@cgnpc.com.cn (S.W.); 3 School of Mechanical and Electrical Engineering, Soochow University, Suzhou 215006, China; 4 College of Mechanical Engineering, Hunan Institute of Science and Technology, Yueyang 421002, China; zhoubox@sina.com

**Keywords:** laser powder bed fusion, pure tungsten, volumetric energy density, microcracks

## Abstract

Tungsten and its alloys have a high atomic number, high melting temperature, and high thermal conductivity, which make them fairly appropriate for use in nuclear applications in an extremely harsh radioactive environment. In recent years, there has been growing research interest in using additive manufacturing techniques to produce tungsten components with complex structures. However, the critical bottleneck for tungsten engineering manufacturing is the high melting temperature and high ductile-to-brittle transition temperature. In this study, laser powder bed fusion has been studied to produce bulk pure tungsten. And finite element analysis was used to simulate the temperature and stress field during laser irradiation. The as-printed surface as well as transverse sections were observed by optical microscopy and scanning electron microscopy to quantitatively study processing defects. The simulated temperature field suggests small-sized powder is beneficial for homogenous melting and provides guidelines for the selection of laser energy density. The experimental results show that ultra-dense tungsten bulk has been successfully obtained within a volumetric energy density of 200–391 J/mm^3^. The obtained relative density can be as high as 99.98%. By quantitative analysis of the pores and surface cracks, the relationships of cracks and pores with laser volumetric energy density have been phenomenologically established. The results are beneficial for controlling defects and surface quality in future engineering applications of tungsten components by additive manufacturing.

## 1. Introduction

Tungsten and its alloys have the intrinsic physical and mechanical merits of a particularly high atomic number and mass, an ultra-high melting temperature, and excellent resistance to corrosion and heat shock. These properties qualify them as strategic materials in promising applications such as plasma-facing component materials or heat sinks in future nuclear fusion reactors [1,2,3,4], gamma or electromagnetic shielding materials in replace of environmentally hazardous lead in both the military [5,6,7] and civilian medical or aerospace industries [8,9,10]. However, some of the above-mentioned properties cause extremely manufacturing difficulties for tungsten-based components. For example, a high melting temperature indicates that rather large amounts of energy are required to break the atomic forces, while cracks and pores are susceptible to forming during solidification, which is macroscopically reflected by the high ductile-to-brittle transition temperature (DBTT, 200–450 °C). Consequently, the tungsten-based components are usually produced by conventional methods using powder metallurgy by sintering [11,12] or hot isotropic pressing [13,14]. These methods can hardly produce complex-shaped components due to the inability to prepare corresponding complex molds.

In the uprising era of additive manufacturing, because of the characteristics of manufacturing complex structures with high precision based on three-dimensional digital models, many 3D printing techniques, such as laser powder bed fusion (LPBF) [15,16,17,18,19,20,21], electron beam melting (EBM) [22,23,24], or even laser direct energy deposition (LDED) [25,26] and electric wire deposition [27], have been studied on pure tungsten or tungsten alloys. More attention has been focused on fabricating tungsten components with LBPF. There are no doubts that controlling processing defects is the most critical issue for future engineering applications. Guo et al. [15] have studied the effect of volumetric energy density (VED) in LPBF on the fast solidifying procedure and the subsequent defects and mechanical properties in pure tungsten. Both large pores and crack networks have been frequently observed in all samples with varying VED. An optimal VED of 1000 J/mm^3^ has been found to obtain a maximum relative density of 98.4%, whereas microcracks seem to be inevitable. In pure tungsten and its alloys fabricated by LPBF, Iveković et al. [16] have reached similar results. They have used various processing strategies to mitigate the formation of pores and cracks, including alloying tungsten with tantalum to lower the melting temperature, preheating the baseplate to 400 °C (close to the DBTT), and laser scanning strategies of bidirectional scanning with an intra-layer misorientation of 90° or 67°. All these efforts have been made to lower the temperature gradient to relieve thermal stress during solidification. Nonetheless, cracks have always been observed in the form of networks, and the maximum relative density is 94.4% for pure tungsten and 97.5% for tungsten–tantalum alloys. Vrancken et al. [17] have also tried similar crack mitigation strategies of alloying with rare-earth element oxides and preheating the substrate to 500–600 °C in LPBF. The preheating seems effective in fully eliminating microcracks in tungsten samples. Obviously, the addition of oxides does not work well because it has no effect on reducing melting temperatures. Whereas Hu et al. [18] found a positive effect of the addition of Y_2_O_3_ to mitigate cracks by forming low-angle twisted tungsten grains. On the contrary, Müller et al. [19] have tried to preheat the substrate to 1000 °C in pure tungsten, where certain pores and microcracks have still been observed. Wang et al. [28] have experimentally studied the formation of crack networks in tungsten. They reached the phenomenological conclusion that cracks form longitudinal and transverse grain boundaries, which are closely related to temperature gradients. Talignani et al. [29] have made a comprehensive review on this topic, covering the additive manufacturing techniques of LPBF, EBM, and LDED. They finally concluded that the controlling mechanisms of defect formation of pores and cracks should be investigated and correlated with mechanical properties, even though much progress has been made in additively manufacturing refractory alloys.

As reviewed above, pores and cracking still remain dominant challenge issues in the field of LPBF fabricating tungsten. From a scientific point of view, these issues are related to the intrinsic properties of high melting temperatures, high brittleness due to DBTT, etc. Hence, efforts have been made from both intrinsic and extrinsic directions, namely, alloying, high pre-heating temperature, laser scanning strategy, etc. In this study, we tried a novel strategy of small-sized feedstock powder as well as high purity of tungsten under normal processing parameters. In pure tungsten, excluding the alloying effect, mitigation of pores and cracks can be solely related to the purity and processing parameters. The finite element analysis has also been used to exhibit temperature fields during LPBF and guide the optimization of processing parameters.

## 2. Experimental

### 2.1. Feedstock Powder

**Figure 1:**
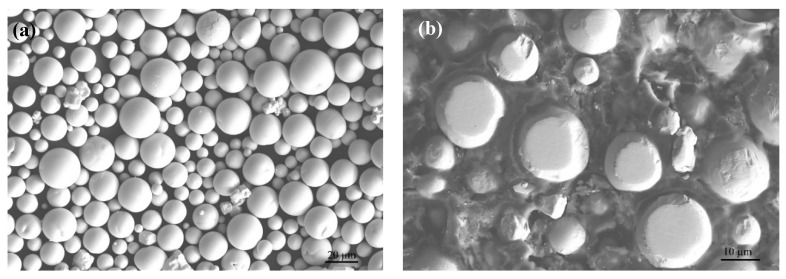
The microstructure of pure tungsten feedstock powder by scanning electron microscopy (**a**) surface morphology; (**b**) cross-sectional morphology.

The tungsten feedstock powder was prepared by plasma spheroidization (Shenzhen Xingchen Inc., Shenzhen, China), with a nominal purity of 99.96% and an oxygen content lower than 200 ppm. The powder showed fairly good spherical shapes and a smooth surface (Figure 1a). Nearly no adhered satellite particles were observed, but there existed some incompletely spheroidized particles of much smaller size, showing irregular shapes. Furthermore, no inner pores were observed from the cross-sectional view (Figure 1b). The powder size was measured in the range of 4.5–26.2 μm, with D10 of 5.1 μm, D50 of 13.9 μm, and D90 of 21.7 μm. The used tungsten powder size was much smaller than that normally used in the LPBF process, which was typically in the range of 5–45 μm.

### 2.2. Laser Powder Bed Fusion Process

The LPBF process was performed with the Suzhou Zrapid iSLM280 system (Suzhou, China). The tungsten sample dimension was designed as 10 × 10 × 10 mm^3^. The laser power was varied between 300 and 380 W, coupled with corresponding scanning velocities of 400 and 600 mm/s. The other key scanning parameters were laser spot diameter of 0.12 mm, powder layer thickness of 0.04 mm, and hatch space of 0.08 mm. The above parameter values result in 12 sample sets, with laser VED varying between 200 and 391 J/mm^3^. The design of the experiment was presented in detail in Table 1. The laser was scanned with a zigzag raster pattern and 67° inter-layer rotation. The oxygen concentration should be well controlled below 200 ppm with flowing 99.999% pure Ar gas during the whole printing process. Otherwise, tungsten tends to form oxides, which are well understood to cause cracks due to their high brittleness. The tungsten plate was used as the building substrate and was preheated to 200 °C.

### 2.3. Microstructural Characterization

**Figure 2:**
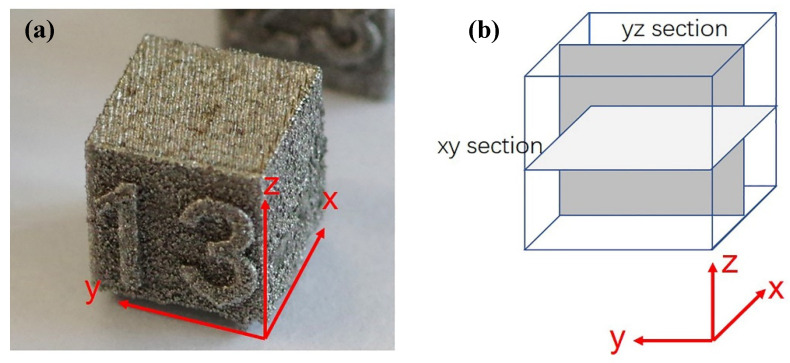
(**a**) The orientation in an as-printed sample and (**b**) the schematic illustration showing the sampling methods of transverse observations.

Figure 2a shows the photo of a typical as-printed tungsten sample, with the xy plane as the scanning plane and z as the build-up direction. The top view on the xy surface was first characterized with scanning electron microscopy (SEM, Zeiss Sigma 300, Oberkochen, Germany) for surface track morphology, and then the cross-sectional view was observed by sectioning parallel to the xy and yz planes almost in the middle position, as schematically shown in Figure 2b. The sectioned samples were mounted, ground, mechanically polished, and etched as per the conventional metallurgical approach.

### 2.4. Finite Element Analysis

**Figure 3:**
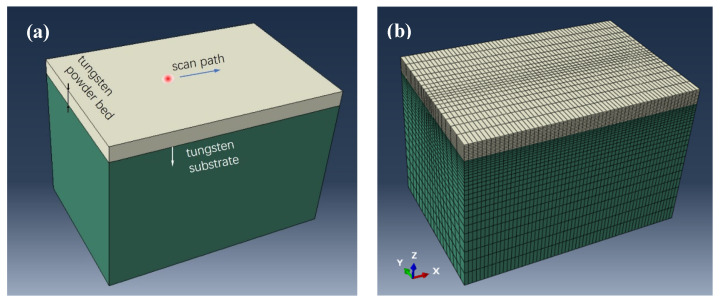
The model in FEA analysis (**a**) and meshing strategy (**b**).

Finite element analysis (FEA) was used to model the thermomechanical behavior as well as the temperature field during a single laser scan track, which could help guide and understand the selection of processing parameters in LPBF processing. A three-dimensional model with a size of 0.6 × 0.6 × 0.4 mm^3^ was constructed with ABAQUS CAE, as shown in Figure 3a. The top layer with a thickness of 0.04 mm was sectioned to denote a tungsten powder bed, and the rest of the lower part was bulk tungsten substrate. The powder bed part was meshed with a size of 0.004 mm, being 1/10 that of thickness and about 1/30 that of laser spot diameter. This can ensure appropriate precision of temperature inside the melt pool. The scan track was aligned on the center surface along the x axis. Thus, the center part and tungsten substrate were double and single bias meshed, respectively, for the tradeoff of calculation accuracy and CPU cost, as shown Figure 3b. The coupled displacement-temperature explicit solver was used for calculation in Abaqus. Thus, the mesh type C3D8RT was chosen for analysis, with 35640 elements in total. The heat source was modeled with the DFLUX user-subroutine, where laser power varied with scanning velocity to obtain high, medium, and low laser VED, designated as H-VED, M-VED, L-VED, respectively (Table 2). The laser power as well as the spot size were also encoded within the subroutine. The real laser spot diameter was used as described in Section 2.2. Considering the surface heat flux, the Gaussian laser beam energy distribution was evaluated according to the following equation, where *P* is the laser power, *α* is the absorption rate of 0.5, *d* is the spot diameter, and *r* is the calculating radius during laser radiation.

$$Q=\frac{4\cdot \alpha \cdot P}{\pi d^{2}}e^{\frac{-8r^{2}}{d^{2}}}$$

The thermo-physical properties of the tungsten substrate and powder bed for FEA modeling were summarized in Table 3 with reference to [30]. A simplified JC model was used for the constitutive mechanical behavior of the pure tungsten in the following equation, where the thermal softening effect was negligible.

$$\sigma =(A+B\varepsilon^{n})(1+C\ln{\dot{\varepsilon }}^{*})$$

Materials constants in the equation are yield stress *A*, strain hardening variable *B*, strain hardening factor *n*, and strain rate hardening variable *C*. In addition, ${\dot{\varepsilon }}^{*}$ is a dimensionless strain rate. These values were extracted from the uniaxial tensile curves in reference [31]: A = 2254 MPa, B = 1264, n = 0.106, C = 0.012, and ${\dot{\varepsilon }}^{*}$ = 1.5.

## 3. Results

### 3.1. Temperature Field in FEA

**Figure 4:**
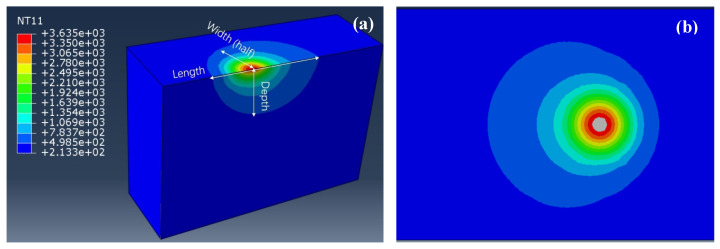
Sectional view of laser beam (**a**) and on the xy surface (**b**) in the case of M-VED.

**Figure 5:**
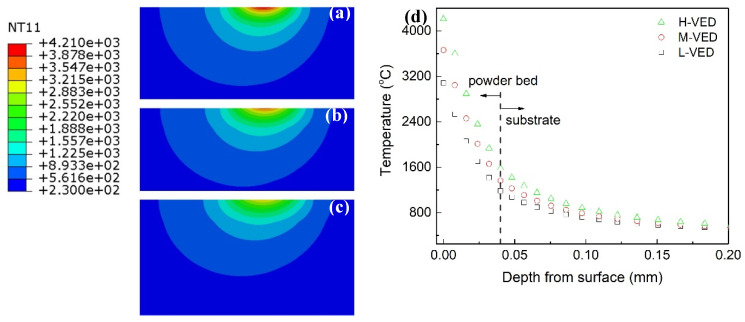
Temperature distribution field along depth in (**a**) H-VED, (**b**) M-VED, and (**c**) L-VED with the same calebar, and (**d**) the comparison of the profiles.

Taking M-VED as an example, Figure 4a shows the typical three-dimensional morphology of the moving laser beam. The size and shape of the laser-heat-affected zone can be observed by sectioning the center part. For the simulated three sets, it seems that the VED has no effect on the dimension of the laser heat-affected size, with a length of 0.26 mm, width of 0.24 mm, and depth of 0.12 mm. With the moving laser spot, the affected zone exhibits a slight elliptical shape, with the aspect ratio of length to width being 1.08. The molten pool size is the critical indicator for understanding the laser–matter reaction during the printing process. If the molten pool is defined as the area where temperature is higher than the tungsten melting point (3422 °C), as shown in the gray area in Figure 4b, the molten pool is dependent on the VED, as presented in detail in Figure 5.

Figure 5 shows the instantaneous node temperature distribution field of the three VED simulation sets. The high, medium, and low VED results in maximum temperatures of 4210 °C, 3661 °C, and 3083 °C, respectively, where the last value is lower than the tungsten melting point. From the depth profiles (Figure 5d) of all three cases, the temperature drops sharply in the powder bed layer (0.04 mm), as guided by the dashed line. Based on estimation from xy planes (Figure 4b as an instance), the molten pool diameter is about 0.04 mm and 0.02 mm for H-VED and M-VED, respectively. Additionally, the corresponding pool depth is only 0.007 mm, 0.004 mm (Figure 5a–d). All these findings suggest that small-sized powder can be heated more homogenously for refractory alloys. The temperature is only about 1380 °C on the powder–substrate boundary. Here, the surface heat flux was used as the heat source. The energy decay along depth was not neglected. This would lead to some errors in estimating the temperature or molten pool size. However, the trend in the three VED variations indicates clearly that sufficiently high laser energy should be imposed to completely melt the tungsten powder. To summarize, the FEA results come up to two points for guiding experimental LPBF processing. First, small-sized tungsten powder is preferred. Second, medium or high VED should be used to obtain a well-formed tungsten component. This helps us design experimental parameters, as shown in Table 1.

### 3.2. Stress Field in FEA

**Figure 6:**
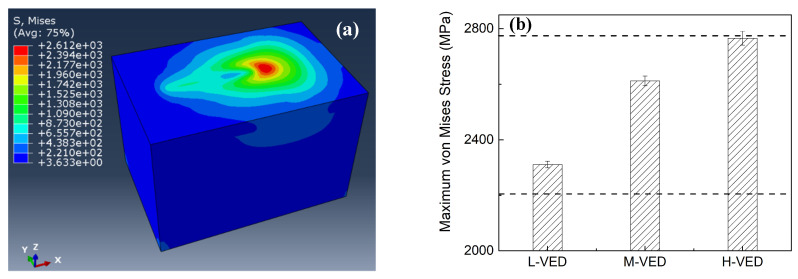
(**a**) The von Mises stress distribution during printing in M-VED sample; (**b**) the maximum von Mises stress in the three cases, the dashed lines indicating the yield and tensile stress of pure tungsten, respectively.

Figure 6 presents a typical von Mises stress distribution during printing in the case of M-VED. Because of the fast heating and cooling in the laser–tungsten interaction, there exists a steep stress field. The maximum stress in M-VED can be as high as 2612 MPa, which is slightly higher than the yield stress (2544 MPa) but lower than the yield stress (about 2783 MPa in [31]). The maximum stress appears on the top surface, decreasing with depth in the powder bed. To exhibit a clearer and more general trend, the maximum von Mises stress at the four calculation timesteps was evaluated for an average value. Figure 6b summarizes the maximum von Mises stress in all the cases with varying VED. In the present simulated processing parameters, no residual stress is higher than the tensile stress of tungsten. However, due to the high cooling rate on the surface, cracks have been indeed observed on surface in experimental results in the following sections.

### 3.3. Surface Morphology

**Figure 7:**
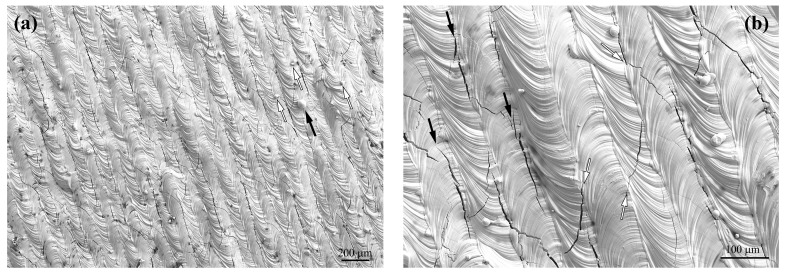
The typical surface morphology of the LPBF tungsten with VED of 291.7 J/mm^3^ on the printed surface xy plane at (**a**) low-magnification and (**b**) high-magnification views.

Macroscopically, all 12 sets of processing parameters result in good-shaped tungsten bulk samples. Taking a VED of 291.7 J/mm^3^ as an instance, Figure 7 presents the typical morphology on the scanning xy plane of the as-printed tungsten sample. In the low-magnification view (Figure 7a), continuous laser scan tracks can be clearly distinguished. The width of the tracks is 73.9 ± 7.9 μm, which is close to the designed hatch space of 80 μm. The surface shows fairly smooth and flat morphology, with nearly no balling or bumping effects being observed. This indicates that the tungsten molten pools are well flattened to make good wetting with each other. Some residual particles exist, which can be traced to two sources. First, the particles with a bigger diameter and a size of several tens of micrometers (as indicated by the black arrow in Figure 7a) may be rooted in spattering due to the recoil forces. This phenomenon is well known as being dependent on the laser–powder bed interaction. However, this is a rather minor effect on tungsten due to its ultra-high mass density. Only a few such large particles have been observed on the surface. The other types of particles are rather small ones with nanosized diameters, as indicated by the white arrows in Figure 7a. These have been formed by the evaporation and subsequent solidification of rather small tungsten, impurity particles, or oxides. This corresponds to the black fumes, which can be normally observed during printing. The element analysis shows a high oxygen content in such particles [32]. The oxidation of the molten pool deteriorates both the surface tension and thermocapillary convection, which further intensifies the balling tendency [33].

Furthermore, as shown in Figure 7b, a certain density of cracks exists on the surface, and most of them are parallel to the scanning direction (black arrows). And they are located at the scan track boundaries, which are almost the overlapping areas of adjacent scans. Such parallel cracks can be attributed to the complex residual stress field due to the heating–cooling cycles in such areas. On the other side, a few cracks across the scan tracks have also been observed. These cracks are nearly normal for laser spot arcs. These two types of surface cracks are in compliance with many results reported in ref [16,18,21,28], where cracks formed network shapes due to the two types of crossed directions. However, compared to the reported crack networks, the crack density in this work is much lower. As in Figure 7, no crack networks can be clearly observed.

### 3.4. Cross-Sectional Morphology

**Figure 8:**
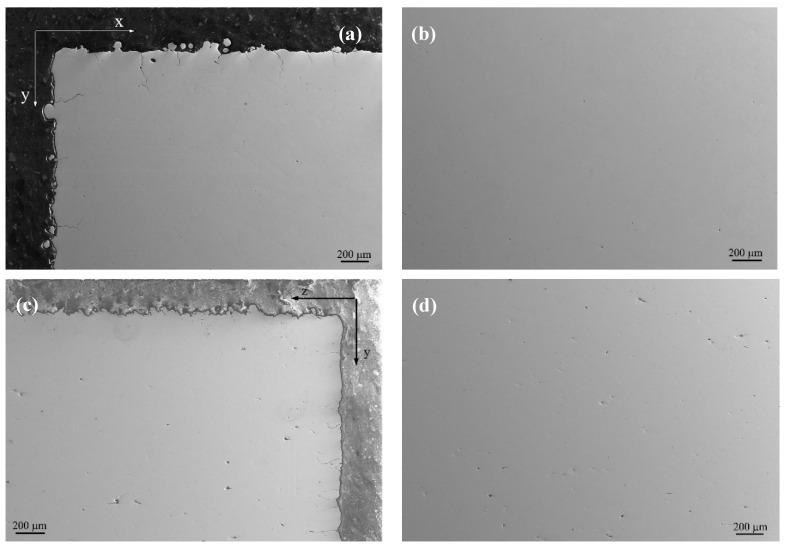
The typical transverse morphology of the polished LPBF tungsten with VED of 291.7 J/mm^3^ and xy and yz sections: (**a**) edge and (**b**) center areas of xy section; (**c**) edge and (**d**) center areas of yz section.

All 12 samples show similar morphology in cross-sectional views, with varying crack or porosity statistics. Figure 8 presents the typical morphology of the as-polished and as-etched samples with a VED of 291.7 J/mm^3^. Viewed from the xy section (Figure 8a,b), a few cracks are observed along both the x and y directions. The cracks originated from the surface and extended to a certain depth into the inner areas, with a crack length in the range of 80–300 μm. This value is several times that of the scan track width (80 μm), indicating that the cracks can transmit across several scans. During the printing process, a larger temperature gradient emerges on the surface than the inner part, which leads to higher residual stress during solidification. The surface crack seems to be inevitable due to the high DBTT in tungsten. If evaluated in terms of numbers per unit distance, the crack density is about 2.5–4.8 /mm along the x direction and is almost similar along the y direction, being 1.9–5.8 /mm.

However, no cracks can be observed in the inner area of the xy section (Figure 8b) due to the low cooling rate and low temperature gradient. Some pores do exist with spherical and elongated shapes. The former is formulated by the unescaped inert shielding gas during printing, while the latter is attributed to the unmelt particles due to low VED locally. It has been statistically estimated that the porosity varies between 0.02 and 0.22% with varying VED. However, cracks have been observed on the printing surface (Figure 7). The reason there are no cracks inside can be attributed to the laser remelting or reheating effect [28]. The subsequent laser scans play a role in the healing effect, which leaves no cracks in the inner part.

On the yz section, cracks can only be observed in the y direction, with a bit higher density of 3.8–7.6 /mm than in the x or y direction in xy section (Figure 8a). No cracks are observed in the z direction since z is the building axis. This indicates that bonding has been well-metallurgically established between adjacent layers. In the inner part (Figure 8d), only pores are observed with a higher porosity percentage than the xy section, being 0.24–1.02%.

**Figure 9:**
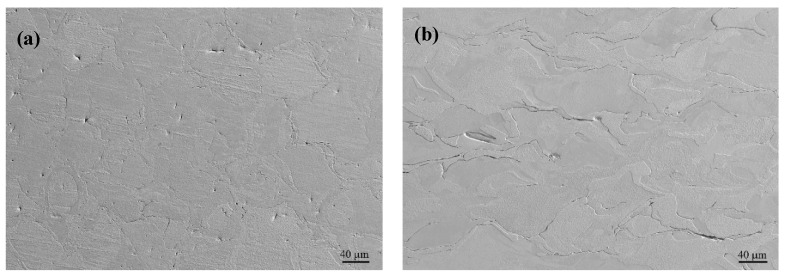
The as-etched morphology of center areas on the section with VED of 291.7 J/mm^3^: (**a**) xy section; (**b**) yz section.

Figure 9 shows the etched morphology with a VED of 291.7 J/mm^3^ in the inner part of the xy and yz sections. On the xy section, the grains exhibit almost equiaxed shapes, with an average grain size of 58 ± 12 μm. The grain boundaries have been more seriously etched than those inside the grains. On the contrary, the grains are elongated in the yz section. This indicates the grains grow preferentially along the laser scan direction, which is the heat dissipation path.

## 4. Discussion

As is widely recognized, the intrinsic properties of high melting temperatures and high DBTT hinder the processing of high-quality tungsten components by LPBF. Too many processing parameters complicate optimizing LPBF processing tungsten. Apparently, an appropriate FEA approach will accelerate the procedure and help understand the laser–matter interaction. In this work, FEA analysis reveals two aspects for guiding experimental designs. First, the maximum temperature in the molten pool indicates a range of combinations of laser power and scanning speed. With M-VED and H-VED resulting in high enough temperatures in FEA, the laser power and scan speed have been experimentally designed in the range of 300–375 W and 400–600 mm/s, respectively. All 12 sets with the combination result in high-quality bulk samples. Second, the FEA reveals a rather small molten pool in tungsten. This justifies our choice of small-sized tungsten powder (diameter of 4.5–26.2 μm) as well as small layer thickness (40 μm). The small size guarantees that the whole layer can be fully remelted during laser irradiation. The sufficient laser energy absorption also lowers the dynamic viscosity of the molten pool. A significant drop in viscosity can be expected with an increase in molten pool temperature [34]. The coupled displacement-temperature solver also helps understand the residual stress. Since tensile data for tungsten can be hardly obtained, the JC model has been simplified, and no damage model has been provided in FEA. Thus, no cracking behaviors can be found in FEA, which is contradictory to the experimental results.

**Figure 10:**
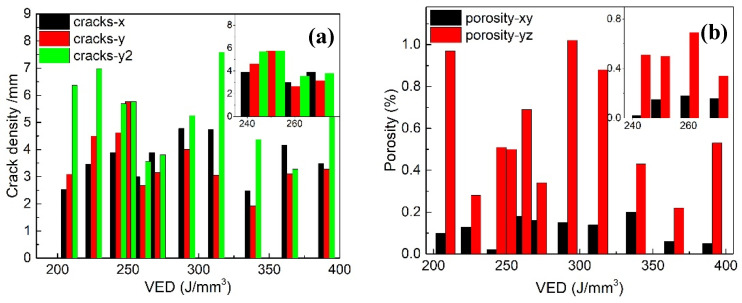
The relationship of defects with laser volumetric energy density: (**a**) crack density; (**b**) porosity.

**Figure 11:**
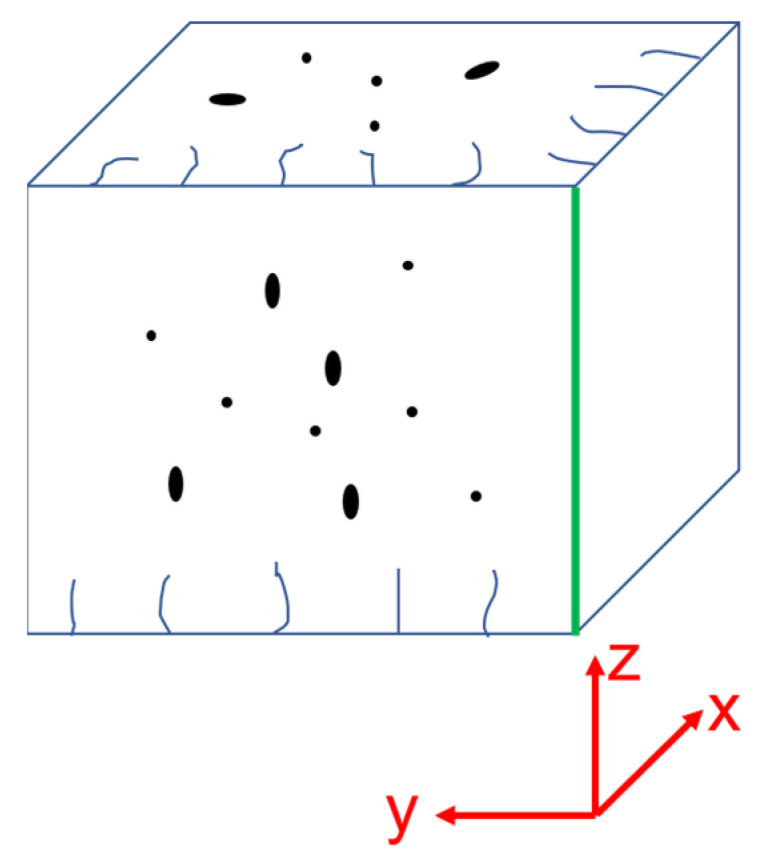
The phenomenological understanding on the defects in the LPBF tungsten.

To understand the cracking behaviors in LPBF tungsten, a phenomenal relationship has been constructed by statistical analysis. For the 12 printed tungsten samples, the varying coupled parameters of laser power and scan velocity can be unified into the laser VED. Figure 10 shows the relationships of crack density and porosity with VED on the xy and yz sections; the inset subfigure magnifies data in the VED range of 235–275 J/mm^3^. In Figure 10a, y2 indicates the crack density along the y direction on the yz section. Combining qualitative (Figure 7 and Figure 8) and quantitative (Figure 10) analysis, the defects of cracks and pores in printed tungsten can be phenomenologically reconstructed in Figure 11.

Porosity is strongly related to processing conditions. With varying densifying strategies like alloying, high preheating temperatures, and optimized scan routes, the lowest porosity in tungsten and its alloys can be in the range of 0.8–4.6% in the published literature [15,16,18,21,34]. Such efforts also help mitigate cracking. In this work, the porosity of the printed tungsten samples is varied between 0.02 and 1.02% with varying VED, corresponding to a relative density of 89.98–99.98%. However, due to such inherent features as high melting temperatures and high DBTT, highly dense bulk tungsten is rather difficult to obtain by additive manufacturing techniques. In addition, research found that it is rather easy to form crack networks with crack parallel and vertical to laser scan tracks, as in [16,18,21,28]. The cracks may spread to the length of several layers along the building direction. In comparison, a simple strategy of small-sized powder and highly pure, highly dense tungsten bulk with a low density of cracks only on the surface has been successfully obtained. The small size effect has been discussed with the FEA molten pool size. The purity, especially oxygen content, has been well controlled within the powder processing and printing chamber. For commercial printers, the oxygen can be easily controlled below 200 ppm, as in this work. However, this is at an economic cost of consumption with highly pure inertia Ar flow. Otherwise, the oxidation of tungsten would inhibit laser energy absorption and cause a high tendency for brittleness. In addition, an oxygen-active atmosphere like hydrogen can also be used during printing. They react with oxygen more easily instead of tungsten.

With varying VEDs of 200–391 J/mm^3^, in this work, highly dense pure tungsten bulk samples with low porosity and only surface cracks have been obtained in LPBF. People have been using high preheating temperatures or alloying strategies to mitigate cracks. It has been known that the mechanism for mitigating cracks relies on the input laser VED. Too much energy will result in a high temperature gradient, whereas too low energy can hardly fully melt metallic powder, especially tungsten with a high melting temperature. The balling effect has been normally observed in low-energy regimes. Meanwhile, tungsten is sensitive to being oxidized, which is highly brittle and detrimental to inducing cracking. Thus, in this work, low oxygen content has been controlled in preparing tungsten powder as well as in the whole printing procedure. The small powder size is another critical factor in obtaining high-density printed tungsten. For a smaller-sized powder, a smaller VED is needed to fully melt tungsten. This leads to a low temperature gradient in the later solidifying process. The FEA-simulated temperature and stress field also support the selection of small-sized tungsten powder. Thus, this work reveals that it can yield fairly acceptable results by using small-sized feedstock powder with high purity, particularly low oxygen content.

## 5. Conclusions

With FEA simulation and systematic experimental study on the LPBF processing of pure tungsten, highly dense bulk tungsten has been successfully fabricated using small-sized feedstock powder and well-controlled oxygen content. The main conclusions have been obtained as follows:

(1) The FEA simulation reveals an appropriate processing parameter window and justifies the selection of small-sized powder, guiding fast experimental design by a relational trend of maximum temperature and stress in the molten pool with the critical processing parameters.
(2) The experimental results prove that highly dense bulk pure tungsten samples can be obtained by processing with LPBF by using powder sizes in the range of 4.5–26.2 μm with low oxygen content. Such samples have a high relative density of 89.98–99.98% under a wide processing VED range of 200–391 J/mm^3^.
(3) Low densities of microcracks have only been observed on the top and side surfaces. The pores and microcracks have been phenomenologically understood, and quantitatively correlated with laser VED. The consequent laser scan heals the inner cracks by remelting or heating them.

## Figures and Tables

**Table 1:** Design of experiment for fabricating tungsten samples by LPBF.

No.	11	12	13	21	22	23	31	32	33	41	42	43
P (W)	300	300	300	325	325	325	350	350	350	375	375	375
V (mm/s)	400	500	600	400	500	600	400	500	600	400	500	600
VED (J/mm^3^)	312.5	250	208.3	338.5	270.8	225.7	364.6	291.7	243.1	390.6	312.5	260.4

**Table 2:** The processing parameters for FEA modeling of temperature field in LPBF.

	Laser Power (W)	Scanning Velocity (mm/s)
H-VED	350	400
M-VED	300	500
L-VED	250	600

**Table 3:** The thermo-physical properties of tungsten powder and substrate used for FEA.

	Density (kg/m^3^)	Specific Heat (J/kgK)	Thermal Conductivity (W/mK)
Tungsten substrate	16,900	209	97.1
Tungsten powder bed	10,100	209	38.6

## Data Availability

The original contributions presented in the study are included in the article, further inquiries can be directed to the corresponding author.
